# Supplementary material for: Design and Preclinical Validation of an Anti-B7-H3-Specific Radiotracer: A Non-Invasive Imaging Tool to Guide B7-H3-Targeted Therapies
Source: Pharmaceuticals (Basel). 2025 Sep 30;18(10):1477. doi: 10.3390/ph18101477 (PMC12567163; doi:10.3390/ph18101477)
Supplement: Supplementary file 1 [file pharmaceuticals-18-01477-s001.zip › pharmaceuticals-3728835-supplementary.pdf]

Design and preclinical validation anti-B7-H3 specific tracer: A non-invasive imaging tool to guide B7-H3 targeted therapies.

Cyprine Neba Funeh[1*]. Fien Meeus[2]. Niels Van Winnendael[1]. Timo W.M. De Groof[1]. Matthias D'Huyvetter[1#]. Nick Devoogdt[1#*]

Supplementary Sheet

1. Supplementary results

Table S1: High-throughput ELISA on 3 B7-H3 recombinant proteins and flow cytometry analysis on B7-H3$^+$ LN-229 cells using periplasmic extracts of 81 first-generation sdAbs. Results are reported as ratios (optical densities of sdAbs divided by blank for ELISA and mean fluorescence intensity (MFI) of sdAbs divided by unstained control for flow cytometry). Ratios of > 2 are considered positive. Highlighted clones are the 13 selected for production. purification. and further characterization. SdAbs are arranged per CDR3 family.

SdAb number	CDR3 family	Periplasmic extract ELISA (optical densities)				ELISA optical density ratio			Ratio MFI
		h4IgB7-H3	h2IgB7-H3	m2IgB7-H3	Blank	h4IgB7-H3	h2IgB7-H3	m2IgB7-H3	LN229 cells
Nb13	1	0.9	0.3	0.2	0.1	6.7	2.6	1.7	4.0
Nb14	1	1.7	0.7	0.4	0.1	12.6	5.3	2.8	6.9
Nb31	1	0.4	0.3	0.3	0.2	1.8	1.2	1.2	9.2
Nb33	1	0.5	0.3	0.3	0.3	1.9	1.1	1.2	2.3
Nb37	1	1.3	0.5	0.3	0.2	8.2	3.2	2.1	0.2
Nb40	1	1.5	1.0	0.5	0.2	9.6	6.4	3.4	1.1
Nb43	1	2.1	1.2	0.7	0.2	11.1	6.2	3.6	6.0
Nb45	1	1.5	0.6	0.3	0.2	10.1	3.7	2.2	11.3
Nb54	1	0.7	0.3	0.2	0.2	4.5	1.8	1.5	6.3
Nb56	1	1.3	0.4	0.3	0.1	9.7	3.4	2.3	5.7
Nb57	1	1.0	0.4	0.3	0.1	7.6	2.9	2.1	1.8
Nb58	1	1.9	0.9	0.6	0.2	10.9	5.1	3.4	7.2
Nb59	1	1.6	0.6	0.4	0.1	11.4	4.1	2.7	27.7
Nb61	1	2.2	1.0	0.7	0.3	8.2	3.7	2.6	17.6
Nb62	1	1.2	0.5	0.4	0.3	4.9	2.1	1.5	3.2
Nb63	1	2.2	1.4	0.8	0.2	12	7.7	4.4	7.3
Nb65	1	1.3	0.5	0.4	0.3	4.5	1.9	1.5	7.4
Nb66	1	1.2	0.5	0.4	0.2	5.2	2.1	1.7	5.0
Nb67	1	0.9	0.4	0.3	0.2	3.6	1.7	1.4	5.6
Nb68	1	2.1	1.3	0.9	0.2	11.7	7.2	4.8	8.0
Nb71	1	1.5	0.5	0.4	0.2	6.2	2.2	1.9	9.3
Nb72	1	1.2	0.5	0.4	0.3	4.5	2.0	1.5	4.9
Nb74	1	0.9	0.4	0.4	0.2	3.7	1.7	1.5	4.1
Nb76	1	1.4	0.6	0.4	0.3	5.6	2.2	1.7	9.8
Nb80	1	0.9	0.5	0.4	0.3	3.3	1.6	1.4	6.1
Nb81	1	2.0	1.4	0.7	0.2	9.5	6.7	3.5	5.1
Nb41	2	1.0	0.4	0.2	0.2	6.1	2.2	1.4	0.9
Nb44	2	2.1	0.8	0.4	0.2	14.2	5.4	2.8	6.5
Nb46	2	1.2	0.5	0.4	0.2	5.2	2.3	1.6	3.4
Nb47	2	1.0	0.3	0.3	0.1	7.2	2.5	2.2	1.7
Nb49	2	0.9	0.4	0.3	0.1	6.4	2.4	1.8	4.8

Nb52	2	1.2	0.5	0.3	0.2	5.5	2.2	1.5	5.1
Nb55	2	1.2	0.4	0.4	0.2	7.4	2.5	2.2	7.9
Nb64	2	1.4	0.5	0.4	0.3	5.1	1.8	1.7	3.4
Nb69	2	2.3	0.8	0.5	0.2	9.6	3.2	2.2	7.8
Nb77	2	2.9	1.1	0.8	0.3	9.8	3.7	2.6	7.9
Nb78	2	2.0	1.4	0.7	0.2	8.7	6.0	3.2	7.6
Nb39	3	1.0	0.4	0.3	0.1	8.1	3.4	2.1	4.4
Nb48	3	0.9	0.7	0.5	0.2	5.6	4.4	3.1	1.3
Nb50	3	1.9	0.7	0.4	0.1	13.5	4.7	2.7	9.5
Nb53	3	2.9	1.2	0.9	0.2	17.5	7.6	5.3	50.3
Nb73	3	1.1	0.6	0.5	0.3	3.6	1.9	1.5	2.0
Nb38	4	0.6	0.5	0.5	0.2	3.8	3.3	3.4	1.9
Nb42	4	1.0	0.9	0.6	0.2	5.7	5.1	3.6	1.6
Nb51	4	1.2	0.9	0.6	0.2	6.5	4.7	3.1	1.8
Nb79	4	0.7	0.3	0.6	0.2	3.7	1.9	3.1	1.5
Nb70	5	1.7	0.8	0.8	0.3	6.1	3.0	2.7	5.6
Nb75	5	0.7	0.3	0.4	0.2	3	1.5	1.9	10.9
Nb60	6	1.2	0.4	0.3	0.2	7.0	2.6	1.6	1.6
Nb7	7	1.0	0.6	0.5	0.2	5.9	3.9	3.0	1.2
Nb8	7	0.5	0.5	0.4	0.2	3.2	3	2.6	1.1
Nb10	7	0.5	0.3	0.2	0.2	3.2	1.7	1.5	1.4
Nb11	7	1.0	0.4	0.3	0.2	6.9	2.5	2.1	1.4
Nb12	7	0.7	0.6	0.4	0.2	4.0	3.4	2.1	1.2
Nb18	7	1.4	0.5	0.4	0.2	7.2	2.4	1.8	1.5
Nb19	7	1.0	0.4	0.3	0.2	5.6	1.9	1.7	1.3
Nb20	7	0.7	0.5	0.3	0.2	4.2	3.1	2.1	1.3
Nb21	7	1.1	0.9	0.5	0.2	5.5	4.5	2.7	1.7
Nb23	7	0.8	0.6	0.4	0.2	4.3	3.5	2.5	1.2
Nb24	7	0.7	0.3	0.2	0.2	3.7	1.6	1.3	1.9
Nb26	7	1.7	1.3	0.7	0.2	11.5	8.3	4.9	1.2
Nb34	7	1.3	0.5	0.4	0.3	4.8	1.7	1.6	2.2
Nb36	7	1.8	1.3	0.8	0.2	7.8	5.5	3.4	0.2
Nb1	8	1.3	0.4	0.4	0.1	9.1	2.7	2.6	1.5
Nb2	8	1.7	0.5	0.4	0.1	12.4	3.7	3.3	11.5
Nb3	8	0.7	0.6	0.4	0.2	4.4	3.8	2.4	1.1
Nb4	8	0.2	0.5	0.4	0.2	1.3	3.4	2.8	1.2
Nb6	8	0.8	0.6	0.5	0.1	5.1	4.2	3.6	1.5
Nb22	8	1.0	0.3	0.3	0.1	7.0	2.4	2.4	2.0
Nb25	8	0.7	0.4	0.4	0.2	4.3	3.0	2.7	1.0
Nb32	8	0.5	0.4	0.4	0.3	1.7	1.2	1.3	1.8
Nb16	9	1.7	0.5	0.5	0.2	10.7	3.4	2.9	2.0
Nb27	9	2.2	0.8	0.6	0.3	7.8	2.7	2.1	6.1
Nb28	9	2.4	0.9	0.8	0.4	6.5	2.5	2.1	8.2
Nb35	9	2.3	1.5	0.9	0.2	13.5	8.8	5.2	3.3
Nb15	10	1.8	0.4	1.2	0.2	11.0	2.3	7.1	6.6
Nb29	10	0.7	0.2	0.5	0.3	2.7	0.9	1.9	9.0
Nb30	10	2.1	0.4	1.3	0.3	6.9	1.3	4.2	8.2
Nb5	11	1.2	0.4	0.4	0.1	8.3	2.8	2.6	1.1
Nb9	11	0.7	0.3	0.3	0.2	4.5	2.1	1.9	1.1
Nb17	12	1.3	0.5	0.6	0.2	6.1	2.3	2.9	1.1

Table S2: High-throughput ELISA on 3 B7-H3 recombinant proteins and flow cytometry analysis on B7-H3$^+$ U87-MG cells using periplasmic extract of 37 second-generation sdAbs. Results are reported as ratios (optical densities of sdAbs divided by blank for ELISA. and mean fluorescence intensity (MFI) of sdAbs divided by unstained control for flow cytometry). Ratios of > 2 are considered positive. Highlighted clones are the 3 selected for production. purification. and further characterization. SdAbs are arranged per CDR3 family.

**sdAb Number**	**CDR3** family	**Periplasmic extract ELISA (optical density)**				**ELISA optical density ratio**			**Ratio MFI**
		**h4IgB7-H3**	**h2IgB7-H3**	**m2IgB7-H3**	**Blank**	**h4IgB7-H3**	**h2IgB7-H3**	**m2IgB7-H3**	**U87-MG cells**
C9	1	1.7	0.2	0.2	0.1	17.1	2.1	1.9	2.9
C13	1	2.6	0.4	0.2	0.1	28.4	4.7	2.6	3.2
C14	1	1.2	0.2	0.2	0.1	13.2	2.1	1.8	1.8
C15	1	1.3	0.2	0.2	0.1	15.0	2.4	2.0	2.4
C21	1	1.7	0.2	0.2	0.1	19	2.2	2.0	4.6
C30	1	1.1	0.2	0.1	0.1	11.1	1.8	1.5	3.9
C72	1	1.0	0.2	0.2	0.1	9.8	2.0	1.7	4.8
C74	1	0.6	0.1	0.2	0.1	7.3	1.7	2.0	1.6
C86	1	0.6	0.1	0.1	0.1	6.9	1.6	1.5	1.8
C92	1	1.0	0.2	0.2	0.1	9.8	2.3	1.5	3.2
C1	3	1.3	0.1	0.1	0.1	12.1	1.4	1.4	1.9
C2	4	0.4	0.1	0.2	0.1	4.6	1.4	1.7	1.5
C25	5	1.1	0.2	0.2	0.1	11.9	1.8	1.7	1.6
C4	13	0.5	0.1	0.1	0.1	5.1	1.3	1.2	1.5
C5	13	1.5	0.1	0.1	0.1	16.6	1.6	1.6	4.1
C8	13	1.5	0.2	0.2	0.1	15.0	2.0	1.8	3.0
C10	13	0.6	0.2	0.2	0.1	5.6	1.8	1.4	1.6
C19	13	0.4	0.1	0.1	0.1	4.4	1.3	1.2	1.4
C22	13	3.0	0.3	0.3	0.1	34.6	2.9	3.3	3.2
C26	13	0.9	0.2	0.2	0.1	10.7	1.7	1.8	1.9
C32	13	0.7	0.1	0.1	0.1	8.1	1.3	1.4	2.1
C44	13	2.2	0.2	0.1	0.1	19.6	1.8	1.3	3.6
C66	13	0.9	0.1	0.1	0.1	8.0	1.1	1.1	1.6
C88	13	0.5	0.1	0.1	0.1	4.9	1.3	1.3	2.3
C98	13	0.5	0.1	0.1	0.1	5.3	1.2	1.3	2.5
C113	13	1.0	0.2	0.1	0.1	10.9	1.8	1.4	3.0
C18	14	0.9	0.2	0.1	0.1	10.2	2.0	1.7	1.9
C38	14	1.0	0.2	0.1	0.1	10.8	1.7	1.5	2.8
C42	14	1.1	0.2	0.1	0.1	12	1.8	1.5	2.7
C51	14	3.0	0.2	0.1	0.1	35.5	2.1	1.5	8.9
C53	14	0.9	0.2	0.1	0.1	9.9	1.8	1.3	2.4
C77	14	1.4	0.2	0.2	0.1	16.6	2.5	2.5	2.5
C80	14	1.9	0.2	0.2	0.1	21.2	2.6	2.6	3.4
C89	14	0.8	0.2	0.1	0.1	8.8	1.8	1.6	3.5
C93	14	0.8	0.2	0.1	0.1	8.2	1.7	1.5	4.6
C101	14	0.6	0.1	0.1	0.1	6.5	1.6	1.3	2.1
C102	14	0.6	0.1	0.1	0.1	6.4	1.6	1.3	12.3

Table S3: Kinetics and associated affinities (K_D) of lead sdAbs on 3 recombinant B7-H3 proteins. performed by SPR (k_a: association rate. k_d: dissociation rate. K_D: equilibrium constant). n=2.

	**Human 4IgB7-H3**			**Human 2IgB7-H3**			**Mouse 2IgB7-H3**		
**sdAb**	**k_a (1/Ms)**	**k_d (1/s)**	**K_D (nM)**	**k_a (1/Ms)**	**k_d (1/s)**	**K_D (nM)**	**k_a (1/Ms)**	**k_d (1/s)**	**K_D (nM)**
C51	2.4E+06	5.8E-02	21 ± 1	3.3E+06	7.1E02	20 ± 0	2.6E+06	8.0E02	37 ± 2
C80	1.4E+06	1.1E-01	105 ± 2	1.7E+06	1.6E-01	79 ± 2	2.1E+06	1.9E-01	111 ± 1
Nb44	2.2E+06	2.3E-01	102 ± 9	7.0E+06	5.1E-01	31 ± 11	1.1E+07	9.7E-01	89 ± 1
Nb55	2.9E+06	1.9E-01	70 ± 1	8.8E+06	4.2E-01	38 ± 9	1.2E+07	8.6E-01	80 ± 8
Nb0	2.2E+06	3.7E-02	14 ± 1	8.4E+06	8.5E-01	216 ± 16	3.2E+06	3.4E-02	9 ± 1

Table S4: Radiochemical characteristics of ^{99m}Tc-labeled sdAbs. Radiochemical purities (RCPs) were determined by instant thin layer chromatography (iTLC). after purification. Radiochemical yield (RCY) was calculated by measuring the activity of the end product and expressing it as a percentage of the starting activity. decay corrected. The specific activity was calculated by dividing the activity of the product by the recovered mass of sdAb in the product after purification (n=3).

Radio tracer	Radiochemical purity (%)	Radiochemical yield (%; decay corrected)	Specific activity (MBq/ug)
[^{99m}Tc]Tc-Nb44-His6	99.3 ± 2.0	68.0 ± 19.3	10.6 ± 9.4
[^{99m}Tc]Tc-Nb55-His6	97.6 ± 1.8	60.2 ± 17.2	7.8 ± 5.4
[^{99m}Tc]Tc-C51-His6	98.6 ± 1.0	64.9 ± 3.9	8.1 ± 4.1
[^{99m}Tc]Tc-C80-His6	98.5 ± 1.4	64.3 ± 5.7	9.4 ± 6.6
[^{99m}Tc]Tc-Nb0-His6	97.9 ± 1.2	61.3 ± 10.7	7.9 ± 4.8
[^{99m}Tc]Tc-R3B23-His6	97.8 ± 1.5	64.3 ± 16.3	8.3 ± 5.6

Table S5: *Ex vivo* biodistribution results of [^{99m}Tc]Tc-sdAbs in U87-MG tumor model (n=5) at 1.5 hours post injection (p.i.). All results are represented as mean ± SD of injected activity per gram organ or tissue (%IA/g).

**Organ /tissue**	**[^{99m}Tc]Tc-Nb44** (%IA/g)	**[^{99m}Tc]Tc-Nb55** (%IA/g)	**[^{99m}Tc]Tc-C51** (%IA/g)	**[^{99m}Tc]Tc-C80** (%IA/g)	**[^{99m}Tc]Tc-Nb0** (%IA/g)	**[^{99m}Tc]Tc-R3B23** (%IA/g)
Blood	0.52 ± 0.15	0.63 ± 0.08	0.48 ± 0.09	0.51 ± 0.10	0.86 ± 0.12	0.55 ± 0.10
Lymph nodes	0.52 ± 0.14	0.43 ± 0.06	0.53 ± 0.13	0.55 ± 0.12	0.57 ± 0.56	0.40 ± 0.05
Thymus	0.45 ± 0.14	0.46 ± 0.14	0.97 ± 0.60	0.57 ± 0.29	0.43 ± 0.18	0.27 ± 0.12
Heart	0.28 ± 0.09	0.26 ± 0.04	0.30 ± 0.05	0.34 ± 0.07	0.38 ± 0.05	0.21 ± 0.04
Lung	0.57 ± 0.14	0.65 ± 0.08	0.76 ± 0.14	0.93 ± 0.22	0.98 ± 0.22	0.54 ± 0.08
Liver	0.66 ± 0.14	0.77 ± 0.07	1.12 ± 0.09	4.60 ± 0.38	1.04 ± 0.12	0.72 ± 0.10
Pancreas	0.14 ± 0.04	0.16 ± 0.03	0.27 ± 0.04	0.30 ± 0.03	0.63 ± 0.10	0.19 ± 0.07
Spleen	0.22 ± 0.05	0.25 ± 0.01	0.41 ± 0.07	1.14 ± 0.12	0.44 ± 0.08	0.21 ± 0.02
Right Kidneys	292.76 ± 40.42	214.97 ± 18.30	235.66 ± 43.74	257.43 ± 26.79	269.32 ± 29.06	222.60 ± 195.19

Stomach	0.43 ± 0.17	0.60 ± 0.10	0.68 ± 0.20	0.67 ± 0.36	1.34 ± 0.31	0.66 ± 0.13
Small intestine	0.26 ± 0.06	0.30 ± 0.03	0.56 ± 0.19	0.76 ± 0.14	0.60 ± 0.05	0.27 ± 0.04
Large intestine	0.33 ± 0.08	0.34 ± 0.05	0.68 ± 0.35	0.91 ± 0.11	0.59 ± 0.08	0.25 ± 0.04
Ovary	0.55 ± 0.19	0.44 ± 0.20	1.05 ± 0.35	0.95 ± 0.22	0.91 ± 0.24	0.37 ± 0.09
Uterus	0.83 ± 0.50	0.49 ± 0.24	1.05 ± 0.32	0.70 ± 0.19	1.13 ± 0.14	0.50 ± 0.16
Skin	0.89 ± 0.60	0.66 ± 0.07	0.91 ± 0.10	0.80 ± 0.13	1.26 ± 0.20	0.42 ± 0.04
Muscle	0.17 ± 0.07	0.17 ± 0.07	0.19 ± 0.02	0.21 ± 0.12	0.28 ± 0.21	0.32 ± 0.44
Bone	0.17 ± 0.06	0.35 ± 0.21	0.35 ± 0.13	0.77 ± 0.63	0.64 ± 0.28	0.13 ± 0.05
Joint	0.38 ± 0.11	0.4 ± 0.29	0.57 ± 0.19	0.53 ± 0.11	0.71 ± 0.05	0.32 ± 0.08
Brain	0.02 ± 0.01	0.03 ± 0.00	0.03 ± 0.00	0.04 ± 0.01	0.04 ± 0.01	0.02 ± 0.00
Tumor	1.51 ± 0.31	2.55 ± 0.75	4.96 ± 1.41	2.14 ± 1.59	3.79 ± 0.80	0.42 ± 0.77

Table S6*. Ex vivo biodistribution results of [^{99m}Tc]Tc-C51 and [^{99m}Tc]Tc-R3B23 in 624MEL tumor model (n = 3) at 1.5 hours post injection (p.i.). All results are represented as mean ± SD of injected activity per gram organ or tissue (%IA/g).*

Organ/Tissue	[^{99m}Tc]Tc-C51 (%IA/g)	[^{99m}Tc]Tc-R3B23 (%IA/g)
Blood	0.45 ± 0.08	0.36 ± 0.10
Heart	0.17 ± 0.03	0.12 ± 0.03
Lung	0.44 ± 0.07	0.31 ± 0.04
Liver	0.41 ± 0.04	0.38 ± 0.11
Pancreas	0.12 ± 0.02	0.10 ± 0.03
Spleen	0.17 ± 0.03	0.14 ± 0.03
R Kidneys	143.40 ± 6.01	113.74 ± 4.99
Stomach	0.28 ± 0.03	0.29 ± 0.03
S. intestine	0.18 ± 0.05	0.12 ± 0.02
L. intestine	0.18 ± 0.03	0.13 ± 0.01
Muscle	0.10 ± 0.01	0.09 ± 0.03
Bone	0.20 ± 0.02	0.11 ± 0.03
Tumor	1.44 ± 0.11	0.14 ± 0.03

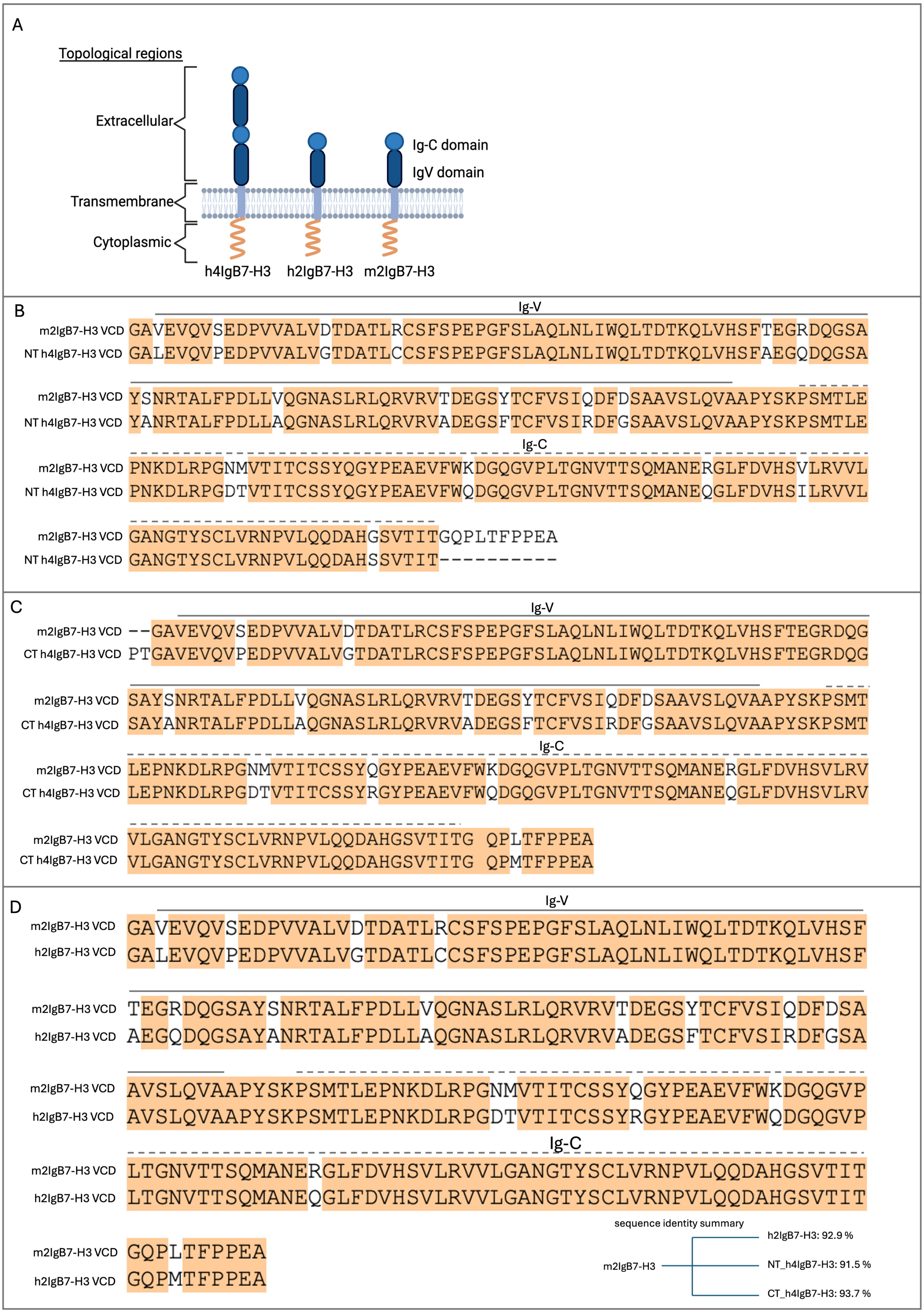

Figure S1: Structural schematics and amino acid sequence alignment of the B7-H3 proteins. (A) Schematics of the B7-H3 proteins showing different topological regions and the IgVC domains. (B-C) m2IgB7-H3

extracellular domain amino acid sequence comparison to the different human B7-H3 isoforms. (B) N-terminal VC domains of h4IgB7-H3. (C) C-terminal VC domains of h4IgB7-H3. (D) h2IgB7-H3 VC domains. Conserved regions are shaded in orange. and unmatched amino acids in white. Solid lines indicate the Ig-V domains. and dotted lines indicate the Ig-C domains. Comparison shows sequence identity between 91% and 94%. Sequence identities are calculated as a percentage of matched identical residues to the total number of aligned positions. NT= N-terminus; CT= C-terminus; VCD= VC domains.

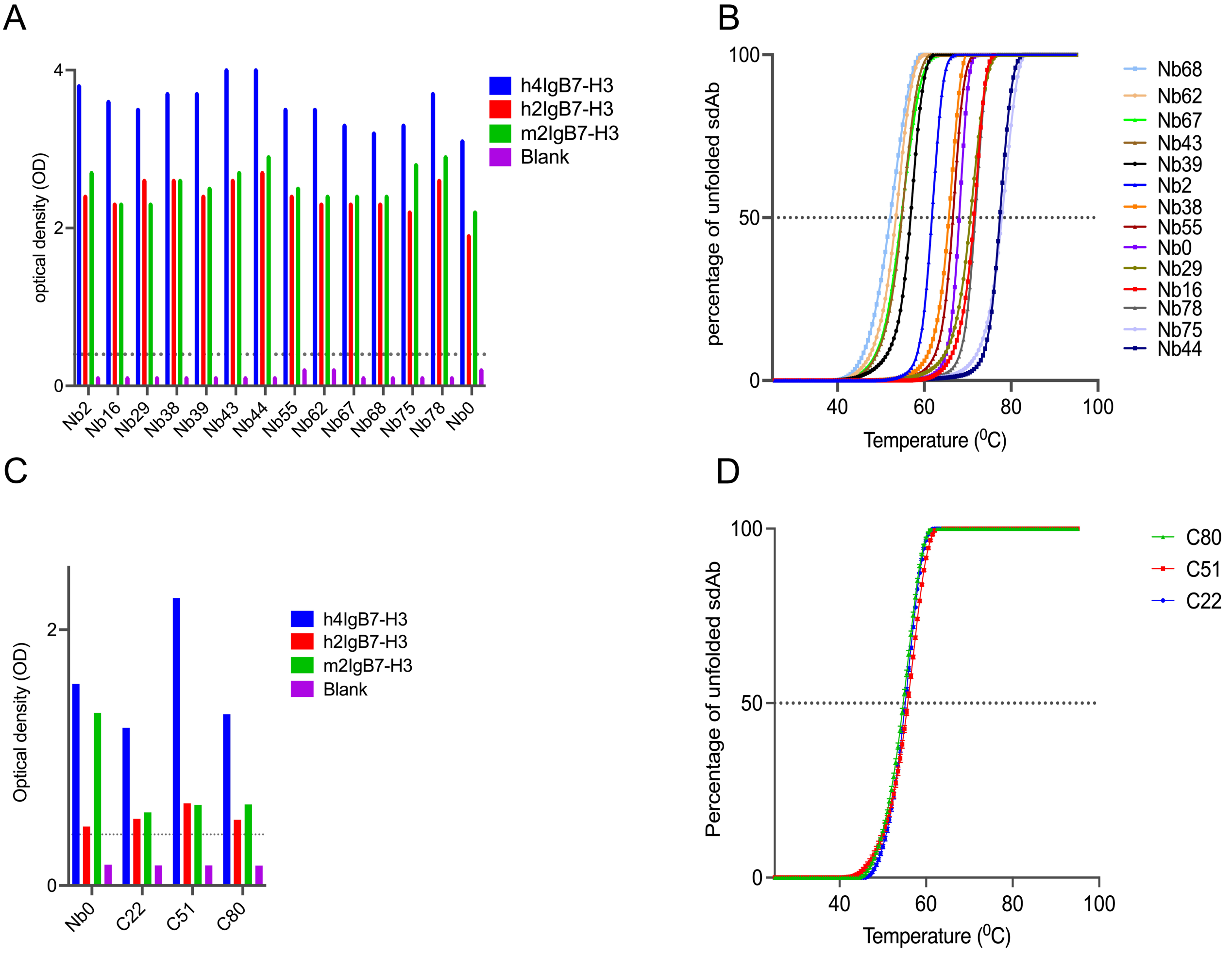

Figure S2. Biophysical and binding properties of purified first- and second-generation sdAbs; (A) Binding ELISA (n=1) for 13 produced first-generation sdAbs on the 3 B7-H3 recombinant proteins. The dotted line represents the cut-off value. An optical density of ≥0.4. 2-fold higher than the blank. was considered reactive. (B) Melting temperature curves of the 13 produced first-generation sdAbs. The melting temperatures were determined as the temperature at which 50 % of the sdAb protein has unfolded (dotted line). Results are represented as mean ± SD. n=3. (C) Binding ELISA (n=1) for 3 second-generation sdAbs using the 3 distinct B7-H3 recombinant proteins. The dotted line represents the cut-off value. An optical density of ≥0.4. 2-fold higher than the blank. was considered reactive. (D) Melting temperature curves for the 3 produced second-generation sdAbs. The experiment was performed and reported as described for (B).

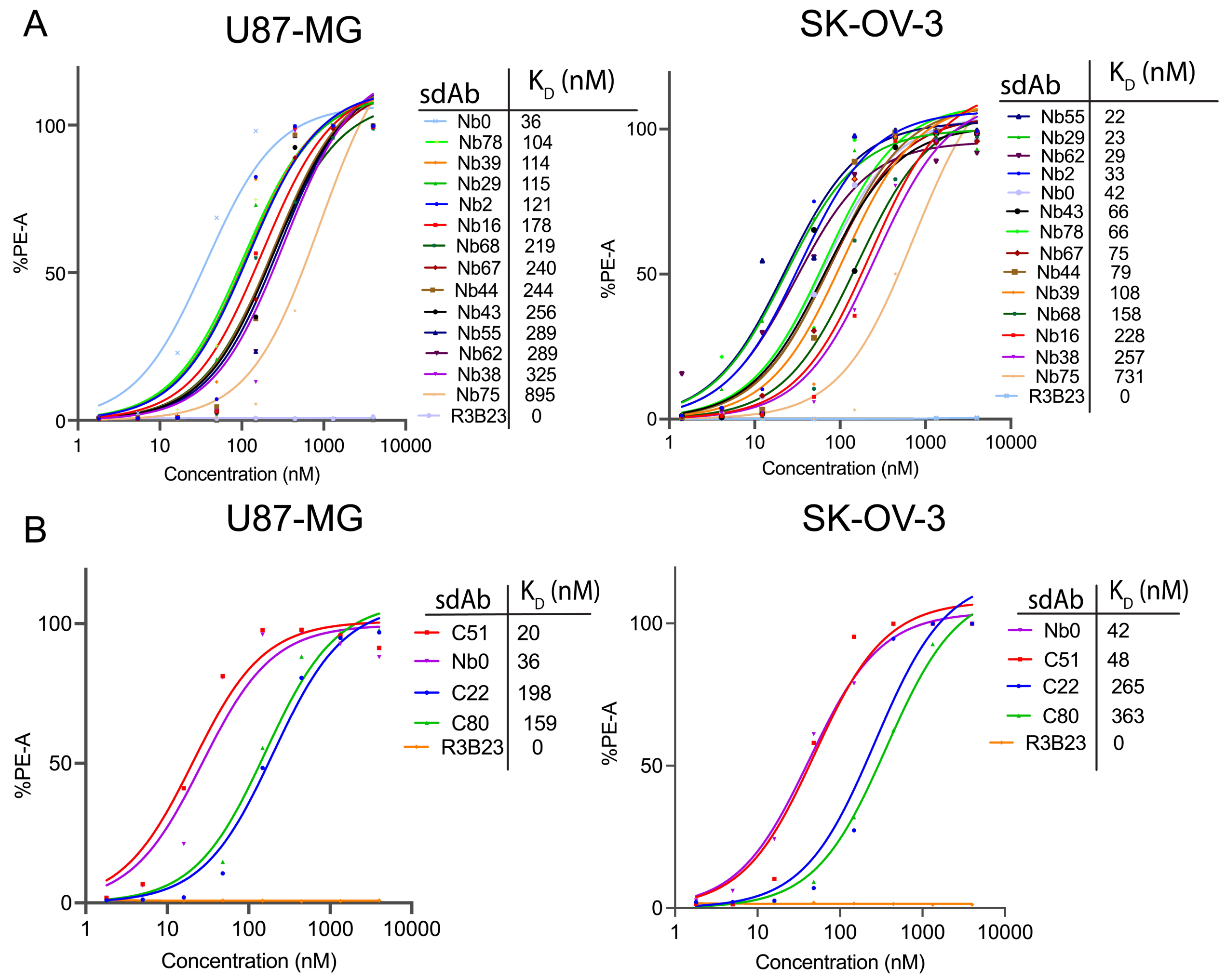

Figure S3*:* Specific binding using a dilution series of anti-B7-H3 SdAbs on U87-MG and SK-OV-3 cells. as assessed by flow cytometry. (A) 13 purified first-generation sdAbs. (B) 3 produced second-generation sdAbs. Curves are plotted according to increasing K_D values of sdAbs. Results are analysed using non-linear regression. n=1.

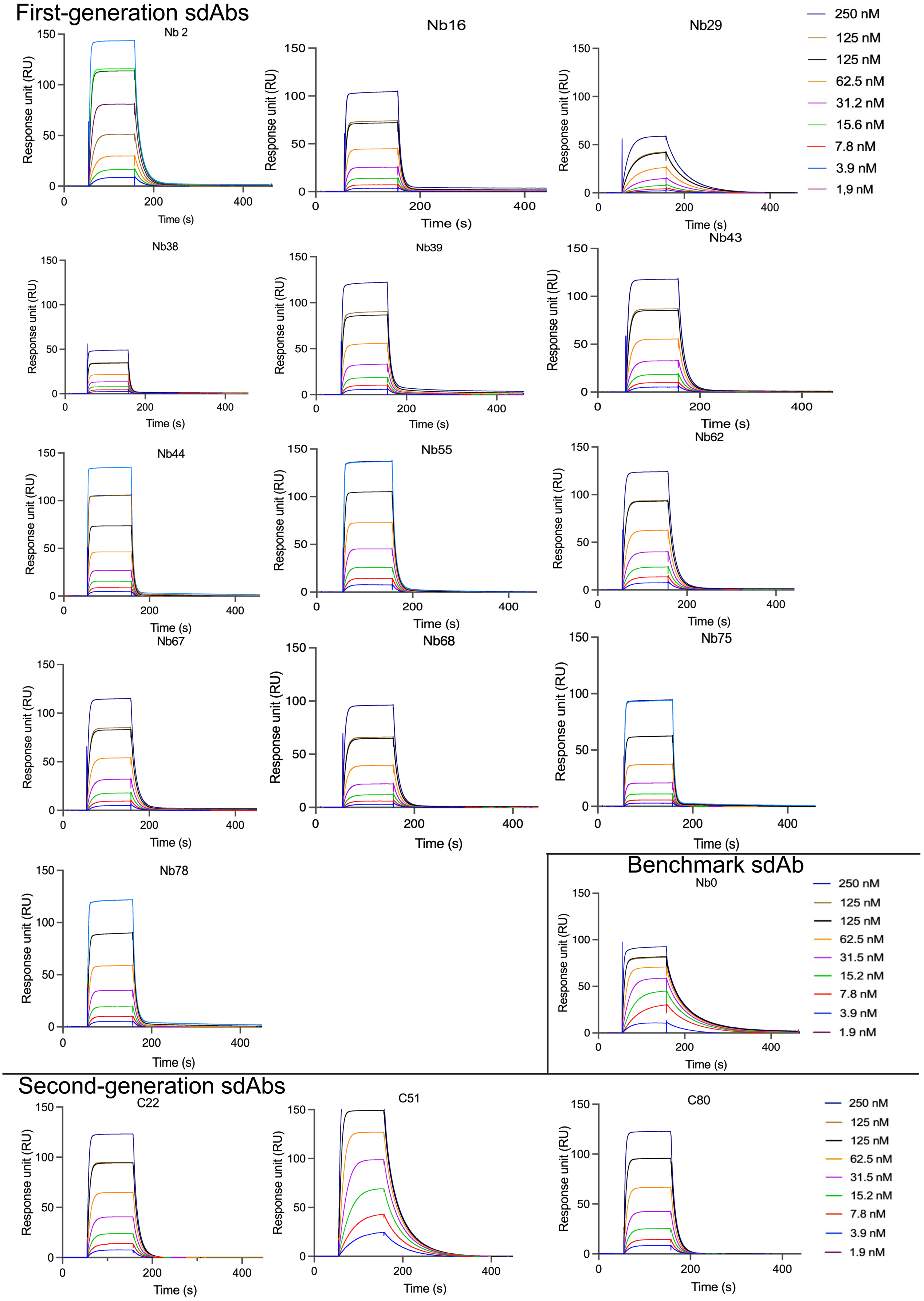

Figure S4: Sensograms of first- and second-generation sdAbs on recombinant h4IgB7-H3 protein as determined by SPR.

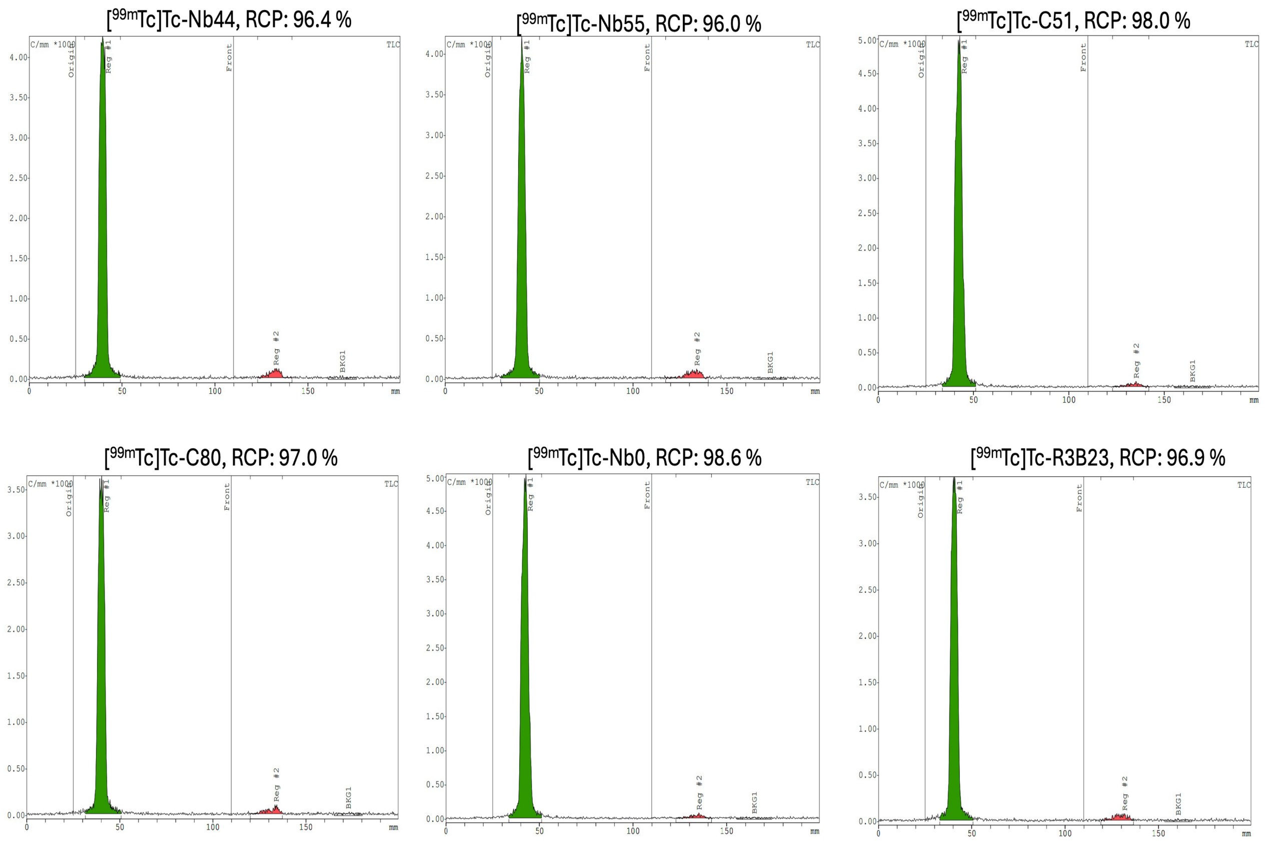

Figure S5: iTLC profiles of ^{99m}Tc-labelled sdAbs. Samples were evaluated using the Elysia Raytest and analyzed with the Gina Star TLC software. Samples with radiochemical purities > 95% were used in animal studies. Region #1 (green) is radiolabelled [^{99m}Tc]Tc-sdAb. and region #2 (red) is uncomplexed or free ^{99m}Tc. (n=7).

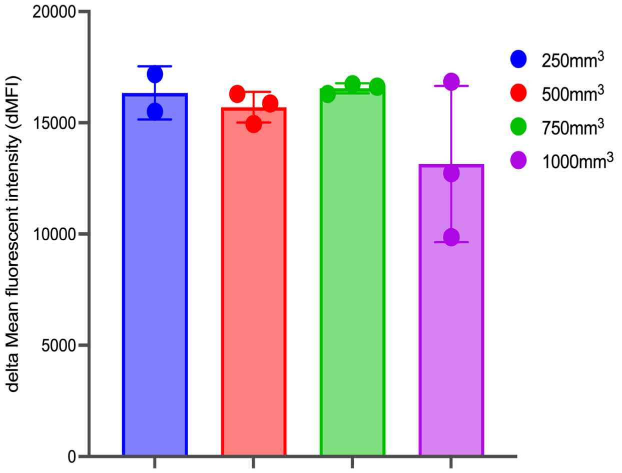

Figure S6: Flow cytometry analysis of human B7-H3 expression on single cell suspensions of U87-MG tumors. Results are shown for different tumor volumes. Stable expression across tumor volumes can be seen. Results are reported as ΔMFI ± SD. n=3.

2. Supplementary materials and methods.

Generation and selection of sdAbs.

Two llamas were subcutaneously inoculated 6 times with 100 µg recombinant extracellular domain of human 4IgB7-H3 protein (Sino Biologicals. Cat. No. 11188-H08H) weekly. supplemented with Gerbu P adjuvant (Gerbu Biotechnik. Heidelberg. Germany). Four days after the last injection. blood was collected. and peripheral blood lymphocytes (PBLs) isolated. followed by the extraction of mRNA from the PBLs. Next. the mRNA was reverse-transcribed into cDNA. and the DNA sequences coding for the variable domain of camelid heavy chain only antibodies were PCR amplified and cloned into pMECS phagemid vectors and transformed into *E. coli* TG1 cells. generating two separate phage libraries from the two llamas. Next. Biopanning was performed by infecting the libraries with M13K07 helper phages. Two generations of biopanning experiments were performed. In the first-generation biopanning experiment. 3 rounds of in-solution panning with each library were performed using the site-specifically biotinylated extracellular domain of h4IgB7-H3 (Avi tag) (Sino Biologicals. Cat. No. 11188-H27H-B). 100 nM of 4IgB7-H3 were used for the panning round 1. followed by 10 nM for rounds 2 and 3.

The second-generation biopanning experiment focused on selecting sdAbs with stronger binding affinities to the B7-H3 protein using stringent conditions. Here. 4 panning rounds were performed with a 10-fold decrease of antigen used for every panning round while increasing the washing interval and volume of washing buffer used. 100 nM of protein was used for the 1st panning round. 10 nM for the 2nd. followed by 1 nM and 0.1 nM for rounds 3 and 4. respectively. For rounds 1 to 3. a 10-cycle washing protocol was used. with the interval between washes progressively increased to 5. 10. and 30 minutes. respectively. For round 4 panning. a 5-cycle washing protocol was employed with a 60-minute interval between each cycle. terminating with an overnight washing step. 1 mL washing buffer was used for rounds 1 and 2. increasing to 2 mL and 5 mL for rounds 3 and 4. Streptavidin-coated magnetic beads (ThermoFisher Scientific. cat 88816) were used to collect the biotinylated antigen/phage complexes. In total. 1050 clones (570 from generation 1 and 480 from generation 2) were randomly selected and screened for their ability to bind specifically to the three isoforms of the B7-H3 protein via ELISA. Positive clones were sequenced and grouped into different families based on their CDR3 similarities. In total. 118 unique clones were identified from both generations.

Production and purification of sdAbs.

Haemagglutinin (HA) C-terminal tagged sdAbs and N-terminal signal sequences were produced in TG1 (*E. coli)* cells transfected with pMECS phagemid vector containing the coding sequence of the

sdAb. After overnight growth in a 2xTY medium. the sdAb expressed in the periplasm was extracted using the freeze and thaw technique as described in [1]. Meanwhile. C-terminal Hexa-histidine (6-His) tagged sdAbs were produced by PCR amplifying the coding sequence of the sdAbs and cloning them into pHEN6 plasmid (mutating the first 5 amino acids (QVQLQ) into DVQLV). using the recombinant cloning technique with the following primers (Fw: 5'-TAC TCG CGG CCC AGC CGG CCA TGG CCG ATG TGC AGC TGG TGG AGT CTG G-3' and Rev: GTG ATG GTG GTG TGA GGA GAC GGT GAC CTG GGT-3'). Next. the pHEN6 plasmid harbouring the sdAb genetic code was transformed into immuno-competent WK6 (*E. Coli*) cells. The bacteria were cultured in supplemented Terrific Broth. and periplasmic expressed sdAbs were extracted by the osmotic shock technique. A benchmark anti-B7-H3 sdAb reported [2] (international patent number PCT/US2019/045918). and an irrelevant sdAb (R3B23) against the multiple myeloma paraprotein 5T2MM [3] were also produced and used as controls in both *in vitro* and *in vivo* experiments. Extracted sdAbs were purified using immobilized metal affinity chromatography (IMAC) HIS-select suspension. followed by size exclusion chromatography (SEC) (Superdex 75 increase 16/600 column. cytiva). with 1X PBS as mobile phase.

Tumor Single-cell suspensions.

U87-MG subcutaneously implanted tumors were harvested and transferred into a GentleMACS C tube (Miltenyi Biotech. Germany) containing 5 mL RPMI medium. The tumors were cut into small pieces of ~5 mm. Next. 150 µL of collagenase I and Dispase II (1000 U/mL) were added. and the tumor pieces were homogenized by incubating in the GentleMACS™ dissociator for 60 minutes at 37 °C. followed by mixing the suspension with 2 µl of DNase I for 37 seconds using the GentleMACS™ dissociator. The suspension was filtered over a 70 µm filter in PBS. followed by centrifugation at 1500 rpm for 5 minutes. Next. red cell lysis buffer (0.16 M NH_4Cl. 0.17M Tris. pH 7.2) was added to get rid of red blood cells. After centrifugation. the supernatant was removed. and the cell pellet was resuspended in RPMI medium.

Next. the cells were initially stained for 20 minutes at room temperature with 1000-fold diluted fixable viability dye (eBioscience™. eFluor™ 506) and 500-fold diluted CD16/CD32 blocking antibodies. After two washing cycles. the cells were stained for 30 minutes at 4 °C with a 1:100 dilution of anti-B7-H3 mAb. and a 100-fold dilution of anti-CD45.2 mAb. An FMO condition stained only with anti-CD45.2 was also included. After two washing cycles. with FACS buffer. data were acquired using a FACSCelesta flow cytometry (BD™ Biosciences) and analysed with FlowJo 10.6.2.

References.

[1] C Vincke. C.. Gutiérrez. C.. Wernery. U.. Devoogdt. N.. Hassanzadeh-Ghassabeh. G.. & Muyldermans. S. (2012). Generation of single domain antibody fragments derived from camelids and generation of manifold constructs. *Methods Mol Biol*. 2012;907:145-176

[2] Robert B. DuBridge. Maia Vinogradova. Ying Zhu. Coexpression and purification method of conditionally activated binding proteins. PCT/US2019/045918. 2021

[3] Lemaire M. D'Huyvetter M. Lahoutte T. Van Valckenborgh E. Menu E. De Bruyne E. et al. Imaging and radioimmunotherapy of multiple myeloma with anti-idiotypic Nanobodies. *Leukemia* 2014. 28. 444–447.
